# Encasing bedding in covers made of microfine fibers reduces exposure to house mite allergens and improves disease management in adult atopic asthmatics

**DOI:** 10.1186/1710-1492-9-44

**Published:** 2013-11-11

**Authors:** Naomi Tsurikisawa, Akemi Saito, Chiyako Oshikata, Takuya Nakazawa, Hiroshi Yasueda, Kazuo Akiyama

**Affiliations:** 1 Department of Allergy and Respirology, National Hospital Organization Sagamihara National Hospital, 18-1 Sakuradai, Minami-ku, Sagamihara, Kanagawa 252-0392, Japan; 2 Clinical Research Center for Allergy and Rheumatology, National Hospital Organization Sagamihara National Hospital, 18-1 Sakuradai, Minami-ku, Sagamihara, Kanagawa 252-0392, Japan

**Keywords:** Adult intervention, Allergen, Atopic asthma, Bed cover, *Dermatophagoides*, Group 1 mite antigen

## Abstract

**Background:**

Studies of avoidance of exposure to group 1 allergens of the *Dermatophagoides* group (Der p 1) have not yielded consistent improvements in adult asthma through avoidance. We explored whether the use of pillow and bed covers and allergen-avoidance counseling resulted in Der 1-level reduction, as measured by enzyme-linked immunosorbent assay, and thus improved asthma symptoms in adult patients.

**Methods:**

Twenty-five adult patients with moderate or severe atopic asthma were randomized into intervention and control groups. Intervention patients slept on pillows and mattresses or futons encased in microfine-fiber covers and were counseled in allergen avoidance through bedroom cleaning. Control patients received neither special covers nor counseling. In the period August to October in 2009 (pre-intervention) and 2010 (post-intervention), dust samples were collected in open Petri dishes placed in bedrooms for 2 weeks and by rapid lifting of dust from bedding and skin using adhesive tape on the morning of 1 day of Petri dish placement. We examined the associations between changes in Der 1 level (as measured by enzyme-linked immunosorbent assay) and clinical symptom score, minimum % peak expiratory flow, and fraction of exhaled nitric oxide.

**Results:**

Der 1 allergen levels on the mattress/futon covers and near the floor of the bedrooms of intervention patients, but not controls, were lower in 2010 than in 2009. From 2009 to 2010, asthma symptom scores decreased significantly, and minimum % peak expiratory flow increased significantly, in intervention patients. The fall in Der p 1 concentration was correlated with a reduction in the fraction of exhaled nitric oxide.

**Conclusions:**

Minimization of Der 1 allergen exposure by encasing pillows and mattresses or futons and receiving counseling on avoiding exposure to indoor allergens improved asthma control in adult patients.

## Background

Exposure of sensitized individuals to allergens, including those associated with house dust mites (HDMs), cats, and fungi, is a risk factor for asthma exacerbation or persistence of asthma symptoms [1-3]. HDMs (i.e., *Dermatophagoides pteronyssinus* and *Dermatophagoides farinae*), are major indoor allergens that can trigger or exacerbate atopic asthma [1-3]. Asthmatic symptoms [4] and bronchial hyper-responsiveness [5] are reduced in children with asthma when exposure to HDMs is minimized by encasing mattresses and using special pillow covers. Individualized, home-based comprehensive environmental intervention can decrease levels of exposure to indoor allergens and reduce asthma-associated morbidity in children [6]. A 90% reduction in allergen levels is feasible and is considered appropriate [1]. However, most studies, including a meta-analysis [7,8], have concluded that reducing levels of exposure to HDMs by using chemical and physical methods does not improve asthma symptoms, levels of medication use, lung function, or the extent of bronchial hyper-responsiveness [7-10]. Particularly in adults, lowering HDM levels does not effectively reduce asthma symptoms [5,11-14]. One report found no clear dose–response relationship between indoor allergen levels and symptom severity [15].

Many factors influence the relationship between exposure to HDM allergens and symptom development or exacerbation. These include the presence of several different allergens on HDMs, the interaction of many factors to enhance the airway inflammatory response, and the existence of several triggers or enhancers of airway narrowing or the perceived severity of symptoms [2]. Such factors include cold air, exercise, passive or active exposure to cigarette smoke and bronchial infection [2]. Unsurprisingly, therefore, several reviews have concluded that avoidance of exposure to HDM allergens is not clinically beneficial for asthma patients and that efforts to avoid these allergens cannot be recommended as part of asthma management [7,8,10]. These results showed that the concentration of *D. pteronyssinus* allergen 1 (Der p 1) in mattresses did not decrease after an intervention treatment by using bed covers, or that lung function in terms of peak expiratory flow (PEF), bronchial hyperresponsiveness, asthma symptom score, and asthma medication use did not change after the concentration of Der p 1 in mattresses was decreased by intervention treatments. In most studies, the level of *Dermatophagoides* mite group 1 (Der 1) allergens in reservoir dust is measured by collecting mites from bedding with a vacuum cleaner and is used as an index of allergen exposure [1,2]. Allergen levels in dust samples are typically presented as micrograms of allergen per gram of dust protein. However, allergen levels in reservoir dust are not necessarily good indicators of the amounts of allergens inhaled [16,17]. Yasueda et al. [18] developed a sensitive fluorometric enzyme-linked immunosorbent assay (ELISA) for detecting Der p 1 and *D. farinae* group 1 (Der f 1) allergens and used it to measure the levels of these components on bedding and human skin. These values were used as indices of exposure and reduced the sensitivity of detection of Der 1 to 1 pg/mL. Earlier, we showed that the levels of Der 1 allergens collected from bedding and skin by using adhesive tape were correlated with those collected from airborne dust through simple dust deposition on plastic Petri dishes [19].

Inhaled corticosteroids (ICS) are the recommended first-line therapy for persistent adult asthma of all grades of severity [20]. In studies of avoidance of mite allergen by intervention using bed covers, almost all adult patients with asthma have been receiving ICS treatment [7].

The above-mentioned earlier measures of allergen levels may not have truly reflected the extent of HDM allergen exposure, and it remains unknown whether measuring Der 1 levels by using the newer, more sensitive, fluorometric ELISA will reveal that exposure avoidance improves clinical symptoms or lung function in adult asthmatics. We explored whether reductions in HDM levels on bedding and in the bedroom, as measured by fluorometric ELISA, affected asthma control in adult patients who were being treated with ICS and other asthma medications.

## Methods

### Patients

Between August and October 2009, we recruited 25 adult asthma patients at the National Hospital Organization, Sagamihara, National Hospital, Kanagawa, Japan. All patients suffered from moderate or severe atopic asthma, as diagnosed by using the criteria of the American Thoracic Society [21]. Asthma severity was assessed by following the current Global Initiative for Asthma (GINA) guidelines [20] and graded accordingly as follows: Step 1, intermittent asthma; Step 2, mildly persistent asthma; Step 3, moderately persistent asthma; and Step 4, severely persistent asthma. All patients had atopic asthma and were sensitized to dust mites, as revealed by measurement of mite-specific IgE. Other allergic conditions exhibited by the patients included allergic rhinitis (which can worsen asthma [22]), allergic dermatitis (diagnosed as described in [23],) and allergic conjunctivitis (diagnosed by using the 2009 criteria of the Japanese Dermatological Association [24]). Exclusion criteria included the presence of pulmonary diseases other than asthma (chronic obstructive pulmonary disease presenting as pulmonary emphysema, interstitial pulmonary fibrosis, or bronchiectasis).

### Study design

Between 1 August and 31 October 2009, each patient collected ambient dust samples in open Petri dishes (plastic, not pre-coated with any protein) (90 × 15 mm; SH90-15; Asahi Glass Co. Ltd., Tokyo, Japan) [19,25,26] that had been left in the bedroom for 2 weeks. Patients also collected bedding and skin dust samples with adhesive tape (Tegaderm Transparent Dressing 1625WJ; 6 × 7 cm; 3 M Health Care, St Paul, MN) [20] on the morning of one day in this period before cleaning the bed room. The patients were randomized into intervention and control groups. Intervention and control patients were classified in two groups composed of odd number and even number in accordance with the highest amount of Der 1 in the bedroom. During February or March 2010, patients in the intervention group were counseled one-on-one once for 30 min in regard to methods of allergen avoidance; the protocol was modified from that of Nishioka et al. [4,27]. In February or March 2010, intervention patients placed covers made of microfine fibers (Microgard; Yasaka Co., Chiba, Japan) over their pillows and futons or mattresses to minimize contact with HDMs. The following recommendations were made. First, all family members’ bedding and futon/mattress covers were to be washed at least monthly at room temperature. Second, the surfaces of mattresses or futons of all family members were to be vacuumed at least weekly with a powerful (>900 W) appliance. Third, wooden floors or tatami matting were to be wiped with a wet cloth before being vacuumed. Fourth, all rooms, including bedrooms, were to be vacuumed at least weekly. Fifth, all carpets were to be removed or (if this was not possible) vacuumed with a powerful appliance at least weekly. Sixth, no stuffed dolls or soft toys were to be kept in the house. Finally, no furred pets were kept in the house. Patients in the control group received neither the special covers nor any hygiene guidelines. In the 2010 sampling period from August to October, a researcher confirmed by interview that all patients in the intervention group were in fact following most of the seven recommended practices described above. All patients collected house dust samples in the period 1 August to 31 October 2010 for follow-up clinical measurement. The start time of the 2-week interval in which each patient collected the 2010 samples differed by less than 1 month from the start time of the sampling interval used by the patient in 2009. Airborne dust was collected in four plastic Petri dishes. Two were placed side by side on the bedroom floor and two were placed side by side at a height of 100 cm above floor level. Both sets of dishes were placed within 2 m of the bed or futon and left undisturbed for 2 weeks before collection. Two adhesive tapes were used to obtain dust samples from the surface of the center of the futon or mattress microfiber cover, where the patient’s back would have been positioned in bed. Similarly, subjects sampled dust on the skin once, on a morning within the sampling period when the bedroom was due to be cleaned, by applying tape to the skin of the right and left neck for a few seconds. All patients continued taking daily ICS; doses, particle sizes, and delivery devices were not changed from 1 August 2009 to 31 October 2010. Other medications taken during the study included long-acting β2 agonists (LABAs), leukotriene receptor antagonists (LTRAs), and long-acting muscarinic antagonists (LAMAs). Clinical symptoms were assessed and used to derive total clinical scores. We documented the presence of cough, sputum production, wheezing, dyspnea caused by an asthma attack, sneezing or nasal discharge attributable to exposure to mites, use of short-acting β stimulants, and emergency hospital visits for asthma treatment for whole period from August 1 to October 31 each year. Symptoms were scored as follows: 0, no occurrence within a month; 1, some occurrences (more than one a month but fewer than two a week); and 2, frequent occurrences (more than once a week).

Our hospital ethics committee approved the study. All relevant tenets of the Helsinki Declaration were followed. Written informed consent was obtained from each patient. The study was supported by Health and Labor Science Research Grants for Research on Allergic Disease and Immunology, awarded by the Ministry of Health, Labor, and Welfare of Japan.

### Measurement of peak expiratory flow

Patients measured PEF three times every morning and evening before bronchodilator use during the 2-week dust collection period each year with an ASSESS PEF meter (CHEST, Tokyo, Japan) [20]; measurements with an error of less than 10% variance among PEF measured three times were recorded by the patient in a diary. After collecting the patients’ diaries we noted the minimum pre-bronchodilator PEF level (as a percentage of the predicted value) obtained within each 2-week test period.

### Measurement of exhaled nitric oxide

NO was measured only once in each test period, on the day on which the Petri dishes were placed in the bedroom. Exhaled air was collected in a Sievers bag that formed part of an NO collection kit (Tsuburai et al. [28]). Each subject took a deep breath of room air through the NO-scavenging filter and exhaled through a mouthpiece with a flow rate of 70 mL/s against an expiratory resistance of 10 cm H_2_O. Five seconds later, the exhaled air was collected into the 1.5-L Mylar bag from the kit. This air was stored at room temperature and the NO concentration measured within 12 h. Air was drawn out of each bag at 200 mL/min into an NO chemiluminescence analyzer (NOA model 280A; Sievers Instruments, Boulder CO) that had a response time of 200 ms. The fraction of expired NO (FeNO) measured by this method was within 80% of that measured by using direct methods [28].

### Eosinophil levels in peripheral blood

We quantified eosinophils and white blood cells (WBCs) in the peripheral blood of all patients by hemocytometry at first hospital visit and at study entry before the intervention.

### Measurement of total IgE, DER F-specific IgE, and DER P 1–specific IgE

Several enzymatic assays employing anti-immunoglobulin E (IgE) antibodies have supplanted the radioallergosorbent test (RAST) [29]. Total IgE levels in serum (IU/mL) were measured by using a radioimmunosorbent test (RIST). Der f–specific IgE (IU/mL) was measured by using crude mite allergen and a RAST that featured the use of ELISA; a nephelometric method was employed (BN II; Dade Behring Inc., Deerfield, IL) [30]. Der p 1–specific IgE levels (UA/mL) were measured with component allergen made by using recombinant antigen and the CAP system (Pharmacia, Uppsala, Sweden).

### Measurement of DER 1 levels

Airborne dust that had settled in the Petri dishes was suspended in 1 mL of phosphate-buffered saline (PBS) with 0.05% (v/v) Tween 20, 0.2% (w/v) bovine serum albumin (BSA), and 0.1% (w/v) sodium azide and then stored at 4°C until analysis of Der p 1 or Der f 1 levels [25]. Samples on adhesive tapes were placed on paper tissue (one sample per tissue) (Kimwipe S-200; Crecia, Tokyo, Japan), placed into polystyrene tubes (10 × 70 mm), and extracted with 2 mL of PBS containing 0.2% (v/v) Tween 20, 0.2% (w/v) BSA, and 0.05% (w/v) sodium azide (PBS-T-BSA) by orbital rotation overnight at room temperature. Dust samples on adhesive tapes at 1:100 w/v (20 mg in 2 mL of PBS-T-BSA) were extracted for 4 h at room temperature. Der p 1 or Der f 1 was quantified by using the fluorometric ELISA developed by Yasueda et al. [18]. Polystyrene microplates were coated for 30 min at 37°C with 200 ng of anti-Der p 1 or anti-Der f 1 monoclonal antibody (P1A03), prepared as described by Yasueda et al. [31]. The plates were incubated overnight at 25°C with 5 ng of biotinylated rabbit anti-Der p 1 or anti-Der f 1 in 100 μL of PBS-T-BSA containing 1 mg/mL normal rabbit γ-globulin. After termination of the reaction with glycin–NaOH, the fluorescence intensity was read with a microplate fluorescence reader (Spectra Fluor; TECAN GmbH, Salzburg, Austria). The excitation wavelength was 360 nm and the emission wavelength 465 nm [18]. Results were expressed as nanograms of allergen per square meter. The Der p 1 or Der f 1 detection limit was 1 pg/mL [18]. Der 1 level was calculated as the total amount Der p 1 and Der f 1.

### Statistical analysis

All values are expressed as means ± 1 SD (with ranges), unless otherwise specified. Statistical comparisons among groups were achieved by using two-way analysis of variance (ANOVA) with a repeated-measures algorithm, followed by post-hoc comparisons using the Newman-Keuls test. The mean values obtained by this process were compared by using the Wilcoxon matched-pairs *T*-test. Correlation coefficients were obtained by using Spearman’s rank correlation test. *P* values of <0.05 were considered statistically significant. Statistical analysis was performed with SPSS for Windows, version 20 (SPSS Inc., Chicago, IL).

## Results

**Figure 1:**
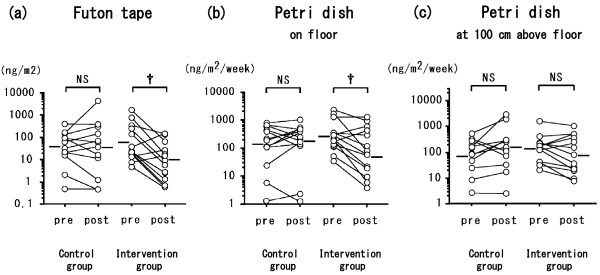
**Der 1 allergen levels (a) on tape samples from futon or mattress covers, (b) in Petri dish samples from the bedroom floor, and (c) in Petri dish samples from the bedroom 100 cm above the floor, for patients in the intervention group before intervention (in 2009) and 1 year later (after intervention), and in the non-intervention group during the same test periods.** Mean values were compared by using the Wilcoxon matched-pairs *T*-test. A *P* value of <0.05 was considered statistically significant. † *P* < 0.01; NS: not significant.

**Figure 2:**
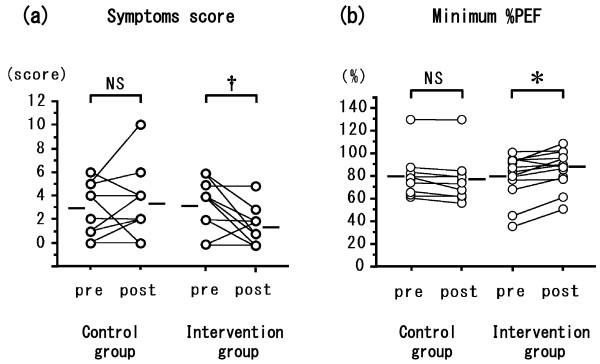
**Clinical symptom scores (a) and minimum % PEF values (b) in patients in the intervention group before intervention (in 2009) and 1 year later (after intervention), and in the non-intervention group during the same test periods.** Mean values were compared with the aid of the Wilcoxon matched-pairs *T*-test. A *P* value of <0.05 was considered statistically significant. * *P* < 0.05; † *P* < 0.01; NS: not significant.

**Figure 3:**
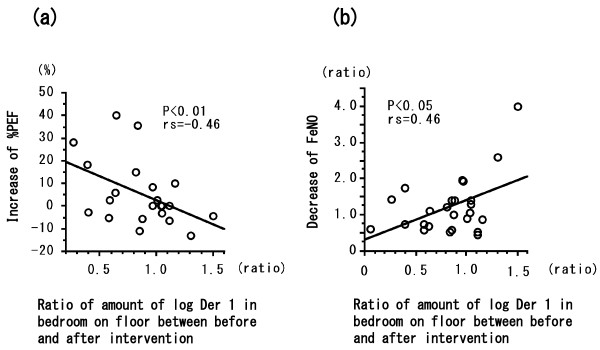
**Correlations between log Der 1 ratio and changes in minimum % PEF value (a) and FeNO level (b) in intervention-group patients before intervention (in 2009) and 1 year later (after intervention), and in the non-intervention group during the same test periods.** Each ratio is [log Der 1 level in 2010 / log Der 1 level in 2009]. The change in minimum % PEF was calculated as (2010*%* PEF–2009*%* PEF)/2009*%* PEF × 100(*%*). Each change in FeNO level was calculated as 2010 FeNO value/2009 FeNO value. Twenty-two of the 25 patients recorded their PEF measurements daily, and 23 of 25 performed FeNO measurements. Correlation coefficients (*r* values) were obtained by using Spearman’s rank correlation test.

**Figure 4:**
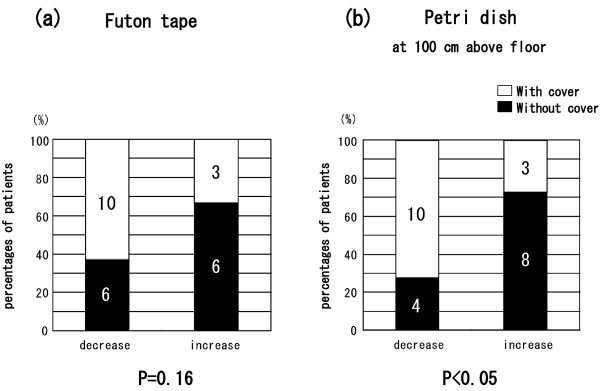
**Decreases or increases in Der 1 levels on (a) tape samples from futon or mattress covers and (b) Petri dish samples from 100 cm above the bedroom floor.** The “increase” and “decrease” groups respectively include data from patients whose 2010 Der 1 levels rose or fell by more than the 2009 Der 1 levels (intervention group = “With cover”; control = “Without cover”). Numbers in boxes are numbers of patients, and y-axes show percentages of patients. The chi-squared test was used to explore the significance of differences between the two groups. A *P* value of <0.05 indicates statistical significance.

**Figure 5:**
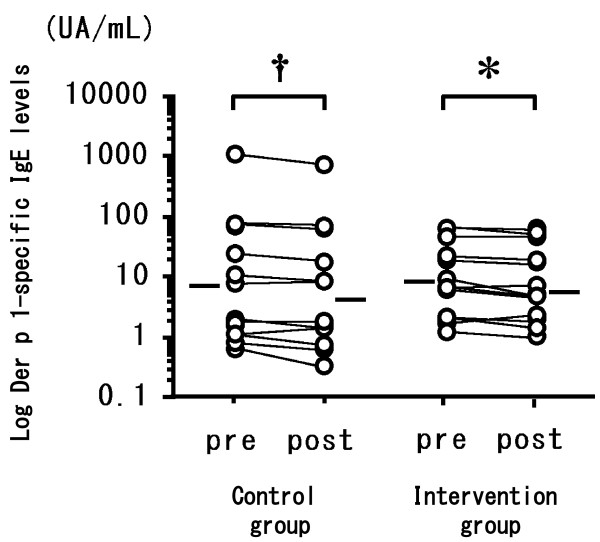
**Log Der p 1–specific IgE levels in patients in the intervention group before intervention (in 2009) and 1 year later (after intervention), and in the non-intervention group during the same test periods.** Der p 1–specific IgE levels (UA/mL) were measured by using the CAP system (Pharmacia, Uppsala, Sweden). Mean values were compared by using the Wilcoxon matched-pairs *T*-test. A *P* value of <0.05 was considered statistically significant. * *P* < 0.05; † *P* < 0.01.

The two groups did not differ significantly in terms of age at entry into the study, sex ratio, age of onset of asthma, duration of asthma, severity of asthma, atopy status, or presence of other atopic disease. All patients were treated with ICS. The groups did not differ significantly in use of the medications LABA, LTRA, LAMA, or theophylline (Table 1). Patients in the intervention group washed their bedding or futon covers, cleaned their mattresses or futons, and wiped surfaces with wet towels before vacuuming more significantly frequently than did controls (data not shown). In the intervention group, one patient kept a cat and three kept dogs; in the control group two kept cats and one kept a dog. No significant between-group differences were evident in terms of serum IgE level (measured by using RIST), IgE RAST for Der f 1, or eosinophil count, either at the time of the first hospital visit or at the time of study entry (Table 2). The eosinophil count in both groups was not higher than the normal range (less than 6% of WBCs). The levels of Der 1 allergen collected from the futon/mattress covers by using tape (Figure 1(a); *P* < 0.01) and from Petri dishes placed on the floor in the bedrooms (Figure 1(b); *P* < 0.01) were significantly lower after intervention; the levels did not differ significantly between pre- and post-intervention in the control group. The levels of Der 1 allergen on the skin tape (data not shown) and in Petri dishes placed 100 cm above the bedroom floor (Figure 1(c)) did not change in either group. The levels of Der 1 on the futon/mattress covers or on skin samples taken with adhesive tape were each significantly correlated with those in the Petri dishes on the floor or 100 cm high (*P* < 0.01; data not shown). The clinical symptom score at the time of assay of Der 1 allergen levels in patients in the intervention group fell after the 2010 intervention (Figure 2(a); *P* < 0.01). The minimum % PEF value in patients in the intervention group increased in 2010 after intervention (Figure 2(b); *P* < 0.05). Neither the clinical symptom score nor the % PEF value differed between 2009 and 2010 in the control patients (Figure 2). FeNO levels did not change between 2009 and 2010 in either group (data not shown). In all patients, the change in Der 1 level in the bedroom in the Petri dishes on the floor between the time of study entry (2009) and the same time in 2010 was inversely correlated with the change in minimum % PEF (*P* < 0.01) (Figure 3(a)) and positively correlated with the change in FeNO level (*P* < 0.05) (Figure 3(b)). However, the change between 2009 and 2010 in Der 1 levels on adhesive tape samples from the skin or bedding was not correlated with the change in minimum % PEF or FeNO level (data not shown). In the intervention group in 2010, Der 1 levels on tape samples from the futon/mattress covers of 10 of 16 patients (Figure 4(a)) (*P* = 0.16) and in the bedroom air of 10 of 14 patients (Figure 4(b)) (*P* < 0.05) fell to lower than the levels before intervention. The levels of Der p 1–specific IgE decreased in patients in both the intervention (Figure 5(a)) (*P* < 0.05) and control (Figure 5(b)) (*P* < 0.01) groups between 2009 and 2010.

**Table 1:** Baseline data on the intervention and control groups

	**Intervention group N = 13**	**Control group N = 12**	***P*-value**
Age (years) at time of study entry; mean ± SD	47.8 ± 11.0	46.5 ± 16.1	NS^1^
Sex (M/F)	5/8	4/8	NS^2^
Atopic rhinitis (yes/no)	9/4	9/3	NS^2^
Atopic conjunctivitis (yes/no)	7/6	10/2	NS^2^
Atopic dermatitis (yes/no)	4/9	5/7	NS^2^
Age at onset of asthma (years); mean ± SD	23.4 ± 19.7	35.5 ± 17.0	NS^1^
Duration of asthma (from time of onset to time of study entry) (years)	25.2 ± 19.9	12.3 ± 13.7	NS^1^
Step 1/2/3/4 asthma severity*	0/2/4/7	0/3/5/4	NS^2^
Daily dose of ICS (μg; converted to CFC-BDP equivalents)	753.8 ± 489.2	566.7 ± 302.5	NS^1^
Use of LABA; n (%)	8 (61.5)	7 (58.3)	NS^2^
Use of LTRA; n (%)	6 (46.2)	5 (41.7)	NS^2^
Use of LAMA; n (%)	2 (15.4)	2 (16.7)	NS^2^
Use of theophylline; n (%)	6 (46.2)	6 (50.0)	NS^2^

Data are presented as means ± SD or means (ranges).

NS, not significant.

^1^ Two-way ANOVA with repeated measures among the two groups.

^2^ Chi-squared testing revealed no significant differences between the values of the two groups.

Values of *P* < 0.05 were considered to be statistically significant.

* According to GINA guidelines.

CFC-BDP, chlorofluorocarbon-propelled beclomethasone dipropionate; LABA, long-acting β2 agonist; LAMA, long-acting muscarinic antagonist; LTRA.

**Table 2:** Serum IgE and eosinophil levels in peripheral blood at the first hospital visit and at study entry

	**Intervention group N = 13**	**Control group N = 12**	***P*-value**
**At first hospital visit**			
Serum IgE RIST (UA/mL)	1718.9 ± 4164.8	928.6 ± 1258.0	NS^1^
Log serum IgE RIST (UA/mL)	2.61 ± 0.69	2.57 ± 0.66	NS^1^
Serum IgE RAST for Der f (IU/mL)	25.3 ± 34.0	28.9 ± 41.4	NS^1^
Log serum IgE RAST for Der f (IU/mL)	1.00 ± 0.72	0.95 ± 0.75	NS^1^
No. of eosinophils (/μL)	430.7 ± 231.4	437.4 ± 315.5	NS^1^
**At study entry**			
Serum IgE RIST (UA/mL)	1169.6 ± 1559.9	589.3 ± 640.0	NS^1^
Log serum IgE RIST (UA/mL)	2.67 ± 0.65	2.57 ± 0.45	NS^1^
Serum IgE RAST for Der f (IU/mL)	24.7 ± 26.2	33.9 ± 48.1	NS^1^
Log serum IgE RAST for Der f (IU/mL)	0.95 ± 0.60	0.89 ± 1.00	NS^1^
No. of eosinophils (/μL)	215.6 ± 149.4	323.8 ± 221.5	NS^1^

Data are presented as means ± SD.

NS, not significant.

^1^ Two-way ANOVA with repeated measures among two groups.

Values of *P* < 0.05 were considered statistically significant.

## Discussion

Levels of Der 1 allergen exposure were decreased in adult patients by encasing pillows and mattresses or futons and giving counseling on avoiding exposure to indoor allergens; asthma control was also increased. Factors that exacerbate asthma in adult patients include not only exposure to indoor allergens derived from HDMs, cockroaches, cats, dog, and fungi, but also respiratory infections [32], development of allergic rhinitis [33], high levels of airborne pollutants [34,35], changes in the weather [36], and exercise [37]. No clear evidence of a dose–response relationship between HDM levels and asthma severity has yet been found [2].

Cockroach allergen (Bla g 1) levels greater than 8 U/g dust are associated with asthma exacerbation. Cockroach allergen avoidance by cleaning, vacuuming, dishes, and sealing food does not reduce asthma exacerbation related to the levels cockroach allergen. Similarly, there is no relationship between cat allergen (Fel d 1) sensitization or symptoms and the current level of cat allergen in the home. Moreover, cat allergen levels in the home for several years are maintained after the cat has been removed [2].

Despite these negative findings, it is possible that reducing Der 1 levels by covering mattresses or futons and cleaning thoroughly in the bedroom may improve the clinical symptoms of asthma [38,39]. In our study, we confirmed that there was no relationship between bedroom Petri dish Fel d 1evels and asthma exacerbation in three patients with asthma and cat allergen sensitivity (data not shown).

Here, we showed that, by avoiding exposure to HDMs in bedding and the bedroom, adult patients with atopic asthma who were sensitive to HDMs achieved total asthma control (as defined by the GINA guidelines [20]), unlike the controls. The levels of Der 1 on the futon or mattress covers of intervention-group patients did not fall upon intervention in 2010 after covers made of microfine fibers were employed, unless these patients followed the directions for allergen avoidance (Figure 4(a)). Thus for a decrease in Der 1 allergen levels the patients needed to not only encase the pillows and futon or mattress cover but also to clean the bedroom as instructed.

Placing bedding in impermeable covers to avoid exposure to indoor allergens has not previously been shown to be of clinical benefit in adult patients [20]. However, such an approach effectively controls asthma in children [40,41]. We found that Der 1 levels in bedrooms were only slightly (but likely valuably) reduced when microfine bedding covers were used (Figure 4(b)). However, the small size of our groups may have influenced the significance of our findings.

Futon or mattress cover levels of Der 1 (sampled by using adhesive tape) were significantly correlated with those in airborne dust (sampled by using Petri dishes) in this study and in another study of ours [19]. The counseling offered to the intervention group likely contributed to the observed reduction in Der 1 levels, because the reduced bedroom air Der 1 level (achieved by applying the recommended cleaning protocols and house hygiene) may have been resulted from the observed reduction in futon or mattress cover Der 1 concentrations. We surmised that the concentration of Der 1 rising up into the bedroom air was decreased not only by the addition of covers but also by vacuuming of the futon or mattress and wiping of the floor with a damp cloth.

Reductions in Der 1 levels on tape samples from futon or mattress covers and in bedroom air samples collected from near the floor over the 2 weeks were associated with improvements in symptom scores and increases in minimum % PEF levels after intervention. However, in the control group neither the ambient level of Der 1 allergen nor the clinical score changed between 2009 and 2010. Reducing levels of exposure to HDM allergens reportedly improves forced expiratory volume in 1 s in asthmatic children [42,43]. However, another study has found that respiratory parameters improve in some, but not all, adult patients [7,44].

FeNO levels did not change between 2009 and 2010 in any of the patients (data not shown). We consider that this was not an artifact caused by the large variation in FeNO levels measured by using our off-line method. Because the observed change in FeNO level after intervention was correlated with the change in bedroom Der 1 level. Reduced exposure to Der 1 caused the FeNO level to fall and the minimum % PEF to improve. Moreover, the extent of airway eosinophilic inflammation may have fallen after intervention, as this is reflected in a drop in mean FeNO values in adult patients with asthma [45].

Most reports and meta-analyses of asthma management have suggested that avoidance of indoor allergen exposure is not of benefit and should not be recommended as a component of asthma management [2,7,10]. However, here we measured Der 1 levels by using a sensitive (1 pg/mL) fluorometric ELISA. Also, it is usual to collect dust samples by vacuuming and to calculate Der 1 levels as micrograms of allergen per gram of dust [1,2,4,27,46]. Instead, we collected dust samples in open Petri dishes and on adhesive tape and detected a few Der 1 level by ELISA above described. We consider that our assay and collection modes were superior to those used in earlier studies; this is likely the reason why reductions in allergen levels were reflected in improved clinical outcomes.

Der p and Der f–specific IgE levels are correlated with allergen exposure among sensitized participants [47]. Interventions that reduce exposure of child asthmatics to HDMs cause the levels of both specific [44,48] and total IgE [49] to fall.

We found, however, that the levels of Der p 1–specific IgE measured with the CAP system fell between 2009 and 2010 in both the intervention and the control group. Thus, reducing the level of exposure to Der 1 did not affect the serum concentration of Der p 1–specific IgE. We consider that the observed fall in reactive IgE levels was attributable to the fact that all patients were treated more intensively (including with ICS) in the interval between the two test periods than was the case before the 2009 test period.

We conclude that precise measurement of ambient Der 1 levels by using a sensitive fluorometric ELISA may be useful in the clinical management of asthma. Also, encasing pillows and mattresses or futons in covers made of microfine fibers and avoiding Der 1 exposure by using an indoor hygiene protocol [4,27] may be of value in adult asthma management.

## Abbreviations

Der 1: *Dermatophagoides* mite antigen group 1; Der p 1: *D. pteronyssinus* allergen 1; Der f 1: *D. farinae* allergen 1; ELISA: Enzyme-linked immunosorbent assay; FeNO: Fraction of exhaled nitric oxide; GINA: Global Initiative for Asthma; HDM: House dust mite; ICS: Inhaled corticosteroids; IgE: Immunoglobulin E; LABA: Long-acting β2 agonist; LAMA: Long-acting muscarinic antagonist; LTRA: Leukotriene receptor antagonist; PEF: Peak expiratory flow.

## Competing interests

The authors declared that they have no competing interest.

## Authors’ contributions

NT examined the patients, analyzed data and statistics, was the main contributor to manuscript preparation, and was involved in manuscript preparation and editing. CO and TN examined the patients and contributed to discussions about the patients. AS assayed the levels of Der 1 on the skin and futon or mattress covers in the bedrooms. YH and KA contributed to discussions about the manuscript. All authors read and approved the final manuscript.
